# Phylogenetic approaches to identifying fragments of the same gene, with application to the wheat genome

**DOI:** 10.1093/bioinformatics/bty772

**Published:** 2018-09-01

**Authors:** Ivana Piližota, Clément-Marie Train, Adrian Altenhoff, Henning Redestig, Christophe Dessimoz

**Affiliations:** 1 Department of Genetics Evolution & Environment, University College London, UK; 2 Department of Computer Science, University College London, UK; 3 Department of Computational Biology, Lausanne, Switzerland; 4 Center for Integrative Genomics University of Lausanne, Lausanne, Switzerland; 5 Swiss Institute of Bioinformatics, Biophore Building, Lausanne, Switzerland; 6 Bayer CropScience NV, Ghent, Belgium

## Abstract

**Motivation:**

As the time and cost of sequencing decrease, the number of available genomes and transcriptomes rapidly increases. Yet the quality of the assemblies and the gene annotations varies considerably and often remains poor, affecting downstream analyses. This is particularly true when fragments of the same gene are annotated as distinct genes, which may cause them to be mistaken as paralogs.

**Results:**

In this study, we introduce two novel phylogenetic tests to infer non-overlapping or partially overlapping genes that are in fact parts of the same gene. One approach collapses branches with low bootstrap support and the other computes a likelihood ratio test. We extensively validated these methods by (i) introducing and recovering fragmentation on the bread wheat, *Triticum aestivum* cv. Chinese Spring, chromosome 3B; (ii) by applying the methods to the low-quality 3B assembly and validating predictions against the high-quality 3B assembly; and (iii) by comparing the performance of the proposed methods to the performance of existing methods, namely Ensembl Compara and ESPRIT. Application of this combination to a draft shotgun assembly of the entire bread wheat genome revealed 1221 pairs of genes that are highly likely to be fragments of the same gene. Our approach demonstrates the power of fine-grained evolutionary inferences across multiple species to improving genome assemblies and annotations.

**Availability and implementation:**

An open source software tool is available at https://github.com/DessimozLab/esprit2.

**Supplementary information:**

Supplementary data are available at *Bioinformatics* online.

## 1 Introduction

Thanks to rapid developments in sequencing technology (reviewed in Goodwin *et al.*, 2016), individual laboratories now routinely sequence and assemble entire genomes and transcriptomes. The most well-established short-read sequencing protocols are cost effective and widely applied. However, without reads that are long enough to span repetitive regions, the assembly step remains a challenge with negative consequences on downstream analyses (Jiao and Schneeberger, 2017; Lee and Tang 2012).

The challenge of genome assembly is particularly acute in plants, which tend to have large and heavily redundant genomes (Claros *et al.*, 2012; Jiao and Schneeberger, 2017). Data from such genomes frequently result in fragmentary assemblies with overestimated gene counts (Denton *et al.*, 2014) and limited utility for downstream purposes such as creation of physical maps used in marker assisted breeding. Fragmentary genes not only lack sequence information—they have been shown to cause problems in downstream analyses, such as in tree inference (Sayyari *et al.*, 2017) and orthology inference (Dalquen *et al.*, 2013; Train *et al.*, 2017).

One problem in low-quality genome assemblies is that fragments of the same gene can be annotated as distinct entries in genome databases; thus such fragments may be wrongly taken to be paralogs. However, it is possible to use homologous proteins conserved in other genomes to detect fragments that are likely to be part of the same gene. To our knowledge, four such approaches have been proposed. First, the Ensembl Compara pipeline (Herrero *et al.*, 2016; Vilella *et al.*, 2009) infers pairs of apparent paralogs as a ‘gene_split’, if they lie within one megabase on the same strand of the same region of the assembly and do not overlap in the multiple sequence alignment of the family. Restricting these predictions to genes belonging to the same contig greatly reduces the risk of false positive split gene calling, but particularly for fragmented assemblies with many short contigs, this approach detects only a fraction of all splits. Second, ESPRIT (Dessimoz *et al.*, 2011) uses pairwise comparisons to identify non-overlapping pairs of paralogs that have a similar evolutionary distance to homologous sequences in other genomes. The third approach, SWiPS (Li and Copley, 2013) is conceptually similar in that it also works based on pairwise alignments—by identifying sets of non-overlapping candidate sequences that have a maximal sum of score with homologous sequences in other genomes. The fourth approach is the computationally efficient PEP_scaffolder (Zhu *et al.*, 2016), which relies on high-identity matches of reference proteins to multiple contigs. Thus, like ESPRIT and SWiPS, the approach relies on pairwise alignments. It also has the strength of considering a maximum intron length to avoid combining gene fragments that are unrealistically far apart.

Yet for all of these methods, the correct identification of split genes heavily depends on their ability to distinguish fragments of the same gene from fragments of paralogous ones. Ensembl Compara and PEP_scaffolder make no attempt to distinguish between the two. As for ESPRIT and SWiPS, although they attempt to identify fragments that match reference proteins consistently—either by requiring consistent evolutionary distances to the reference for all fragments or by requiring consistent best matches for all fragments—these comparisons are inherently limited to pairwise comparison, which loses out on phylogenetic information available in a multiple-sequence and tree setting.

Here, to address this problem, we present two complementary phylogenetic methods to identify non-overlapping or partially overlapping fragments of the same gene that exploit evolutionary relationships across gene families. The first one exploits bootstrap support, and the second relies on likelihood ratio tests. We evaluate their performance on an artificially fragmented version of the reference sequence assembly of the hexaploid bread wheat chromosome 3B (Choulet *et al.*, 2014). We also compare the two methods, and a meta-approach combining the two methods with ESPRIT (Dessimoz *et al.*, 2011), with the Ensembl Compara pipeline and ESPRIT. Finally, we apply new phylogeny-based methods to the early, highly fragmented, draft release of the entire hexaploid wheat genome (International Wheat Genome Sequencing Consortium (IWGSC), 2014) and identify 1221 high-confidence pairs of split genes.

## 2 Algorithm

We first introduce our phylogenetic tests of split genes, then proceed to describe the datasets analysed and the evaluation methods. Note that we provide the fine implementations details in the Supplementary materials.

### 2.1 Phylogenetic tests of split genes

Given a genome assembly with a large number of annotated contigs, the task we face is to figure out which annotated genes actually belong to the same gene, due to annotation mistakes or where the assembler failed to concatenate collinear contigs. Consider therefore two non-overlapping fragments of the same gene sequence. If we perform a multiple sequence alignment of the two fragments together with full-length homologs from other species, and infer a tree based on the alignment, we can expect that the two fragments: (i) align to different regions of the multiple sequence alignment (since they are non-overlapping) and (ii) have a similar evolutionary distance to homologous sequences in other genomes.

However, perhaps surprisingly at first sight, although these fragments will generally be close to one another on the tree, they will almost never be inferred as sister leaves. The reason for this is that since they have no character in common, they cannot be directly compared with each other, only with the other genes in the tree. Thus, there is no phylogenetic information available to infer the relationship between them. The location of the split between the two sequences is therefore undetermined. Furthermore, recall that evolution is modelled as a stochastic process on a tree, with each column in the alignment being a realization of the process. Due to this stochastic nature, the two fragments will almost never attach to the exact same place on the tree.

Under the correct model of evolution, however, if the two fragments originate from the same sequence, the difference in the place these are attached to the tree should not be significant.

Here, we introduce two tests to infer whether two non-overlapping or partially overlapping sequences from the same genome are fragments of the same gene: collapsing branches with low bootstrap support (Efron *et al.*, 1996) and a likelihood ratio test (Wilks, 1938).

### 2.2 Test #1: collapsing ‘insignificant’ branches

Tree branch support measures are commonly used to gauge the reliability of a branch. Since fragments of the same genes can be expected to be separated by insignificant internal branches on the tree, collapsing branches with low support should result in fragments becoming sister leaves. Thus, for a given threshold, the test collapses all branches below that threshold and infers all candidates that are sister leaves as fragments of the same gene.

### 2.3 Test #2: likelihood ratio test

The second test to infer fragments of the same gene is a likelihood ratio test (LRT). Our null hypothesis (labelled ‘s’ for split) is that fragments come from the same gene, and thus can be concatenated into one sequence. The alternative hypothesis (called ‘p’ for paralogs) is that the two fragments belong to paralogous genes.

*H_s_*: *n −* 1 genes (split gene*)*

*H_p_*: *n* genes (paralogous genes)

**Fig. 1.**
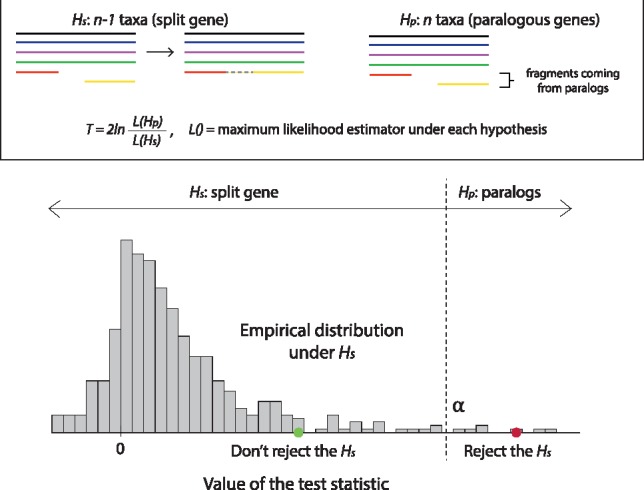
Conceptual overview of the likelihood ratio test. The null hypothesis is that the two fragments come from the same gene (*Hs*) while the alternative hypothesis is that the two fragments come from different paralogous copies (*Hp*). *α* refers to the significance level, which is the area under the curve above the rejection threshold. This setup is motivated by the fact that the split gene hypothesis has fewer parameters. However, it is unusual in that failure to reject the test leads to a prediction, and not the other way round. Furthermore, because the two models are not nested, we estimate the null distribution empirically

The test statistic is defined as $T\;=\;2ln\frac{L(H_{P})}{L(H_{S})},\;$where *L*() denotes the maximum estimator under each hypothesis (Fig. 1).

This setup is unusual in two respects. First, considering that we aim to predict split genes, it may come as a little suspect that we use *H_s_* as null model. Our motivation for this is however that the model with split genes is more constrained (the two fragments sit at the same place in the tree) and thus has fewer free parameters. It is the ‘simpler’ model. For this reason, in absence of evidence to the contrary, we deem it reasonable to favour *H_s_*. Second, in a typical setting of the likelihood ratio test, the null model is a special case of the alternative model. The models are said to be ‘nested’, and theory tells us that the test statistic—twice the difference in log likelihood—is χ^2^ distributed. Since our models are not nested, the distribution of the test statistic given under the assumption that *H_s_* is true is unknown. We can bypass this problem by estimating the empirical distribution under the null using bootstrapping (Efron and Tibshirani 1993; Goldman 1993). Hence, for a particular sample, we:

1. Compute the value of the test statistic; let’s denote it by *T*_0_
2. Since we have no prior knowledge on the distribution of the test statistic under the null hypothesis, we estimate the distribution using non-parametric bootstrapping. First, from the multiple sequence alignment used under the *H_s_*, we generate *n* artificial alignments of the same length, i.e. *n* bootstrap samples by sampling columns with replacement. Second, we create alignments to be used under the *H_p_* by splitting a target *full-length* gene (i.e. the one made up of two candidate fragments) at the same position as in the original alignment. Finally, we compute the test statistic for each of the *n* samples; let’s denote them by T_1_*, T_2_*, _**…**_, T_n_*. If the sampling is correct, the distribution of T_i_*, *i* = 1, 2,_**…**_, *n* will converge to the true distribution of the test statistic when *n***→ ∞**. Hence, if repeated many times, the distribution of the bootstrap sample test statistic values will approximate the distribution of the unknown test statistic. Throughout this project, we set *n* to 100 unless otherwise stated.
3. Compute bootstrap *p* value as the proportion of samples with likelihood equal or above that of the input data: $p_{B}=\frac{\left\{\text{No}.\;\text{of}\;T_{i}\;*\;\geq T_{0}\right\}}{n}$

## 3 Implementation

As input candidate pairs, we identify, among all the protein sequences of a target genome, those that belong to the same gene family—either established by Ensembl Compara or defined as deepest hierarchical orthologous groups (HOGs) as inferred by OMA (Altenhoff *et al.*, 2013). We further require that fragments be non-overlapping, or overlapping with less than 10% residues of both fragment being aligned in the same alignment column, using Mafft v7.164b (Katoh and Standley, 2013). In other words, we require that *a*_12_ < 0.1·*l*_1_ AND *a*_12_ < 0.1·*l*_2_, where *l*_1_ and *l*_2_ are the number of residues in the two fragments, and *a*_12_ is the number of these residues that are aligned. Thus, for each gene family, we align the sequences, enumerate all possible pairs of sequences belonging to the target genome and retain as candidate pairs those that satisfy the aforementioned overlap requirement.

The LRT requires computing maximum likelihood estimates, i.e. finding an optimal tree under both *H_s_* and *H_p_*. Under the *H_s_* hypothesis, fragments are part of the same gene. Hence, in order to find a maximum-likelihood tree under the *H_s_*, we concatenate the candidate fragments into a single sequence. To correct for some cases when a tree-building method gives a suboptimal tree, which may result in the estimated *T*_0_ < 0, we performed two tree searches under the *H_p_* model; a tree search without providing an input topology, and a tree search with an input topology starting with the best tree under *H_s_* with the two hypothetical fragments as sister leaves), and proceeded with the tree with higher likelihood.

Some genes might be involved in multiple predictions, i.e. in more than one pair of fragments coming from a split gene. If all these multiple predictions span different parts of the sequences, we conclude that the gene is split in more than two pieces and consider these predictions as non-ambiguous. If in contrast more than one prediction spans over a common part of the sequence (which might be the case if the fragments come from very closely related paralogs, or if alternative splicing variants of the same gene are erroneously annotated as separate genes), we report the overlapping predictions as ambiguous.

### 3.1 Datasets and evaluation methodology

As a test case for evaluation and application of the methods, we used the proteome of bread wheat, i.e. *Triticum aestivum* cv. Chinese Spring. The bread wheat genome is notoriously large (∼17 Gbp) and redundant: it is a hexaploid genome which arose from two recent allopolyploidization events—with the three subgenomes referred to as A, B and D. Because of this large size and redundancy, the wheat genome is proving very difficult to assemble and annotate. In 2014, the International Wheat Genome Sequencing Consortium (IWGSC) published a highly fragmented chromosome-by-chromosome survey sequence of the bread wheat genome (International Wheat Genome Sequencing Consortium (IWGSC), 2014). The same year, Choulet *et al.* (2014) published a high-quality reference sequence of bread wheat chromosome 3B (third chromosome of subgenome B). The two provide a good basis to evaluate our methodology on a challenging dataset.

We also tested our method on the cassava genome, i.e. *Manihot esculenta*, draft version 4.1 (Prochnik *et al.*, 2012) retrieved from Phytozome v7 (Goodstein *et al.*, 2012).

As customary in the field, we determine the quality of the methods by measuring the precision and recall. Here the recall measures the proportion of fragmented genes that the methods can identify. The precision penalizes for erroneous predictions by measuring the proportion of predictions that are indeed fragmented genes. For both measures, we simulated fragmentation on the wheat 3B reference sequence and cassava genome. In a subsequent experiment, we applied the tests to the wheat 3B survey sequence and validated predictions using the wheat 3B reference sequence. In this case the total number of fragmented genes is unknown, so we could only count the number of correct and wrong predictions, and calculate the precision.

Finally, we applied the methods to the rest of the wheat survey sequence to infer split genes in the bread wheat proteome.

### 3.2 Random fragmentation of the wheat 3B reference assembly (recall)

To determine the recall of the methods, we simulated fragmentation on genes assigned to a high-quality assembly of bread wheat chromosome 3B (3B reference sequence). All genes and their gene families were obtained from Ensembl Plants, release 31. We randomly chose 100 genes, each at least 100 amino-acids long, and split them at a random position such that both fragments are at least 50 amino-acids long. All alignments were performed using Mafft v7.164b with default parameters. Gene trees were built by FastTree v2.1.8 (Price *et al.* 2010), also with a default set of parameters.

In addition, we simulated fragmentation in a more challenging setting, i.e. on small gene families typically containing only evolutionarily very close paralogs. As a source of homologous groups, we used HOGs. They were computed by the GETHOGs algorithm with a default set of parameters on the input dataset comprised of 13 plants: bread wheat and 12 flowering plants exported from OMA Browser (Altenhoff *et al.* 2014) (Supplementary Table S1).

### 3.3 Introducing non-overlapping paralogs in wheat 3B reference assembly (precision)

To inspect cases where the methods incorrectly predict split genes, we simulated fragments from pairs of paralogs assigned to the bread wheat 3B reference sequence using the same datasets as above. We chose 100 pairs of same-species paralogs, cut them at a random position and took two complementary fragments (one from each initial gene) each being at least 50 amino-acids long. Again, MSAs were obtained by Mafft v7.164b (default parameters) and gene trees by FastTree v2.1.8 (default parameters).

Similarly as above, we also simulated more challenging cases of fragmentation. We used the same set of HOGs as in the previous section.

### 3.4 Introducing fragmentation in cassava assembly (recall, precision)

To inspect behavior of the tests on species other than wheat, we introduced random fragmentation in cassava draft proteome (v4.1) downloaded from Phytozome v7. As reference species, we used the 16 other dicot species available in OMA Browser, Dec 2017 release (Supplementary Table S2), of which the closest species is the western balsam poplar, which has diverged from cassava approximately 80 MYA (Fawcett *et al*., 2009). Gene families were again obtained by running GETHOGs algorithm with default settings. Having a set of rather distant species, we proceeded with families containing no more than 100 genes where at least two of them were cassava genes and other genes were assigned to at least nine distinct reference species. To calculate recall and precision, we randomly chose 200 cassava genes and 200 pairs of cassava paralogous genes, each of them being at least 200 amino-acids long, which we fragmented the same way as in the case of wheat. All alignments and trees were reconstructed with the same software and settings as for the wheat.

### 3.5 Validation on 3B survey assembly

To assess predictions on the real data containing fragmented genes, we applied our approaches to a low-quality assembly of bread wheat chromosome 3B, the 3B survey sequence (IWGSP1; 2013-11-MIPS), and compared the predictions with the high-quality assembly of chromosome 3B (‘3B reference sequence’) downloaded from URGI (https://urgi.versailles.inra.fr). As a gold standard, we mapped sequences between the two assemblies using BLAST+ v2.2.30 (Camacho *et al.* 2009).

For the predictions, we used the same reference species as in the simulations on HOGs (see sections 3.2 and 3.3) which we again exported from OMA Browser (Supplementary Table S3). We computed gene families by the GETHOGs algorithm with a default set of parameters. We generated 500 bootstrap samples for each family and performed both tests on fragments overlapping less than 10%. Sequences were aligned with Mafft v7.164b (default parameters) and trees built with FastTree v2.1.8 (default parameters) as above. In addition, we also computed HOGs with a different set of parameters and repeated the rest of the experiment.

For the assessment, the mapping of sequences between the survey and high-quality genomes was not straightforward because the two differ not only in the degree of fragmentation, but also in some of the sequences themselves due to sequencing error, contamination etc. To allow for a bit of tolerance while still maintaining unambiguous mapping between the two, we required hits to cover at least 95% of the corresponding query, the percentage identity in these matching regions to be at least 95%, and the hit to be unambiguous. As a stringent control, we also performed a validation where, in addition to these two requirements, we only allowed mismatches to occur at the ends of a query sequence.

### 3.6 Comparison to established methods and meta-approach

As a point of comparison, we employed the Ensembl Compara pipeline and ESPRIT on the same 3B survey sequence as above. Again, the obtained predictions from each method were mapped to the 3B reference sequence by BLAST+ v2.2.30 to inspect if predicted pairs belong to the same gene or not, requiring both coverage and percentage identity to be at least 95%. Validated predictions were compared to the results from Validation experiment on 3B survey sequence with the same BLAST+ criteria.

To obtain a comparable set of predictions on the 3B survey sequence using public results available from the Ensembl Compara pipeline, we filtered ‘gene_split’ pairs from their homologies file (release 21). We took only pairs where both genes were at least 50 amino-acids long and such that, when its corresponding gene family was aligned with Mafft v7.164b, candidate genes overlapped for less than 10%. We also included cases where more than two genes were inferred as a part of the same gene given that no two genes involved overlapped for 10% or more. Since some of the sequences could not be found in the OMA Browser dataset used for validating Collapsing and LRT approach, we classified Ensembl predictions into two groups: those that could be found in the OMA Browser dataset, and hence, included in the comparison, and those that could not.

Another set of predictions was obtained by running ESPRIT on the same 3B survey sequence data using 12 reference plants (the same dataset as in the Validation section, Supplementary Table S3) keeping all parameters default but increasing the required length of the candidate genes to be at least 50 amino-acids (option ‘MinSeqLenContig = 50’). We only considered a confident unambiguous set of predictions (reported in the *hits.txt* output file).

In addition, we considered a meta-approach ESPRIT 2.0, which encompasses ESPRIT and the new combined approach. It takes the union of predictions made by ESPRIT and our joint method (collapsing branches with support lower than 0.95 and likelihood ratio test with significance of 0.01).

### 3.7 Inferring split genes on the rest of the wheat survey assembly

Finally, we employed the tests to infer fragmented genes in the first draft release of the predicted genes in whole bread wheat genome, i.e. *T. aestivum* cv. Chinese Spring proteome (IWGSP1; 2013-11-MIPS). We considered only candidate fragments assigned to the same chromosome and the same chromosome arm. We used the same reference genomes as in the previous analyses with HOGs (see above). Based on simulations and validation on the 3B survey sequence, we determined a set of parameters used for predictions. In particular, we ran GETHOGs with default parameters and allowed candidate fragments to mutually overlap less than 10% in the corresponding MSA. We used Mafft v7.164b to get alignments and FastTree v2.1.8 to construct trees, both with their default set of parameters. Finally, we chose 0.95 as a threshold for collapsing and set the significance threshold of the LRT to 0.01.

## 4 Results

Recall that we aim to identify fragments of the same gene wrongly annotated as separate genes in a genome of interest, leveraging genomes of related species. In the previous section, we introduced two phylogenetic methods: one based on collapsing branches with low bootstrap support and the other relying on a likelihood ratio test (LRT). To evaluate the methods and determine parameters for predictions on the bread wheat assembly, we took two approaches. First, we simulated fragmentation on the real data to calculate recall and precision. Then, we applied both methods to the bread wheat chromosome 3B survey sequence and validated predictions with respect to the 3B reference sequence. Finally, based on the best parameters obtained from these analyses, we applied the method to infer split genes in the 20 other chromosomes of the survey wheat genome assembly.

### 4.1 Artificial fragmentation of the wheat 3B reference assembly

To assess our methods, we first simulated fragmentation in 100 protein sequences from the high-quality wheat 3B reference assembly and tried to recover these pairs. Our simulations also included 100 pairs of non-overlapping fragments generated from pairs of randomly selected paralogous genes—which can be very difficult negative cases if the paralogs are near-identical.

**Fig. 2.**
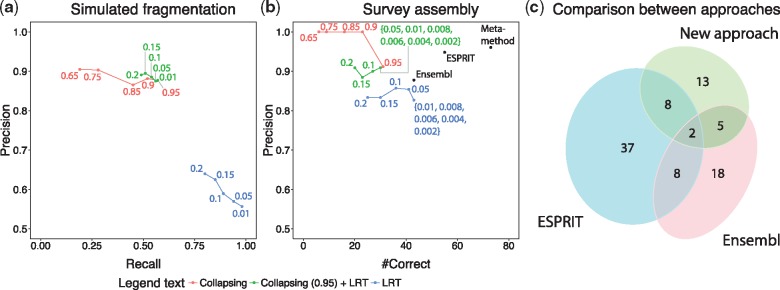
Evaluation of the methods. (**a**) Wheat genes from the high-quality wheat 3B chromosome were artificially fragmented and recovered by the collapsing, likelihood ratio test (LRT) and a combination of the two. Numbers indicate the threshold used for each datapoint. (**b**) Split genes inferred on the low-quality (‘survey’) wheat genome were validated using the high-quality wheat 3B, and comparison with three other approaches (Ensembl Compara, ESPRIT and the meta-method). Numbers indicate the threshold used for each datapoint. The meta-method takes union of ESPRIT’s and the predictions inferred when combining collapsing approach (threshold 0.95) and LRT (significance 0.01). (**c**) The number of predictions on 3B survey sequence classified as correct in the BLAST+ validation. ’New approach’ denotes a combination (intersection) of collapsing approach (threshold 0.95) and LRT (significance 0.01)

On these challenging simulations, the collapsing test yielded high precision (0.85–0.88) and moderate recall (0.20–0.58), while the LRT performed the other way round, yielding moderate precision (0.56–0.64) and high recall (0.81–0.99) (Fig. 2a;Supplementary File S1, Supplementary File esprit2_simulations.tar.gz).

We also evaluated an approach that combines our two methods. A split gene was inferred if both methods were in agreement. This approach resembled the recall and precision of the collapsing approach (with the same threshold) but with slightly higher precision (Fig. 2a, Supplementary File S1).

As a control, we performed another set of simulations using a different set of input homologous sequences—OMA HOGs containing protein sequences from thirteen plants including wheat (Supplementary File S1, Supplementary File esprit2_simulations.tar.gz). Precision of the collapsing test was again high (0.73–0.81) while recall varied between 0.30 and 0.78. Precision of the LRT was moderate to high (0.51–0.89) and the recall was high (0.70–0.75) (Supplementary Fig. S3a). As additional controls, we also repeated the analysis by changing one parameter of the pipeline at a time:

1. alignment mode in Mafft that relies on local alignments, which could conceivably deal better with fragments (L-INS-I instead of FFT-NS-2)
2. special mode in FastTree that reportedly deals better with fragmentary genes (option ‘-pseudo’)
3. increasing the number of bootstrap replicates from 100 to 500 in the likelihood ratio test
4. increasing the number of artificially introduced split genes from 100 to 500.

All these variants yielded qualitatively similar results (Supplementary Fig. S4).

### 4.2 Artificial fragmentation of the cassava genome

To assess whether our approach also works on a different set of species, we performed the artificial fragmentation analysis on 200 single and 200 paralogous pairs of randomly selected the cassava genes. As reference species, we used the 16 other dicot species available in OMA version December 2017, of which the closest species is the Western balsam poplar, which has diverged from cassava approximately 80 MYA (Fawcett *et al.*, 2009).

Using the combined approach of collapsing (threshold 0.95)+LRT(significance 0.01), we observed a recall of 63% and a precision of 40% (Supplementary File esprit2_simulations_cassava.tar.gz). Inspection of the false positives revealed that nearly all mistakes were due to fragmentation of close (i.e. species-specific) paralogs, which to our method are indistinguishable from split genes. Thus, the lower precision is explained by the much higher frequency of species-specific paralogs in cassava (75% of sampled paralogs in cassava versus 13% in wheat). Indeed, when we repeated the analysis excluding artificial fragmentation of such species-specific paralogs, the precision increased to 65%, in line with the results on wheat (Supplementary Fig. S4c).

### 4.3 Validation on 3B survey assembly

To further assess the tests and identify suitable parameters, we applied our methods on the chromosome 3B of the draft-quality bread wheat survey genome (International Wheat Genome Sequencing Consortium (IWGSC), 2014). We focused on this chromosome arm because it was one for which a much higher-quality reference assembly was available, obtained through painstaking piecewise sequencing and assembly using 8452 bacterial artificial chromosomes (Choulet *et al.*, 2014). To give an idea of the improvement between the two, the N50 statistic, which measures the assembly quality by reporting the minimum contig/scaffold length needed to cover 50% of the assembly, is 2.7 kb for the draft genome 3B chromosome versus 892 kb for the high-quality 3B chromosome. Thus, the latter could be treated as ground truth—thus enabling us to gauge, in a realistic setup, how well our approaches can infer split genes in a highly fragmented genome with abundant potential paralogs.

Overall, the methods achieved higher precision than when applied to simulated fragmentation (Fig. 2b). The analysis showed particularly high precision with the collapsing approach. The absolute recall rate could not be easily assessed on these real data; instead, we considered the number of correctly predicted HOG annotations as a surrogate for recall, yielding results highly consistent with the simulations (Fig. 2b).

One challenge with this setup was the fact that the draft survey sequence assembly contains other types of problems, such as sequencing errors or ∼10% contamination from other chromosomes (International Wheat Genome Sequencing Consortium (IWGSC), 2014). If we only consider fragments that can be perfectly mapped between the draft whole-genome assembly and the reference assembly (no mismatch in their central part, see Supplementary materials), the number of predictions that could be validated diminishes, but on the remaining set, our approaches showed even higher precision (Supplementary Fig. S3c and d), indicating that the performance reported in Figure 2b is conservative.

Control experiments also gave consistent results (Supplementary File S2, Supplementary File esprit2_validation.tar.gz). As expected, relaxing parameters yielded more predicted split genes, but at a cost of lower precision (Fig. 2b versus Supplementary Fig. S3b).

### 4.4 Comparison to established methods and meta-approach

To gain further insights into the performance of the proposed approaches, we compared them to two existing methods, namely Ensembl Compara pipeline (which however cannot easily be run on custom genome data) and ESPRIT, as described in Section 3. Both methods were applied to the 3B survey sequence and then validated against the 3B reference sequence using BLAST+ (Supplementary File esprit2_comparison.tar.gz). We also considered a meta-approach, which we call ESPRIT 2.0, comprising ESPRIT and a combination of the collapsing approach (threshold 0.95) and LRT (significance 0.01).

In terms of the number of correct predictions, Ensembl Compara and ESPRIT performed equally well or better than our approaches displaying high precision (Fig. 2b and Supplementary Table S4). Further analysis showed that predictions from different methods are rather complementary and worthwhile to take into account (Supplementary Fig. S5). Hence, the meta-approach, ESPRIT 2.0, inferred by far the biggest number of correct predictions with high precision (Fig. 2b and Supplementary Table S4).

### 4.5 Predictions on the rest of the survey assembly

**Fig. 3.**
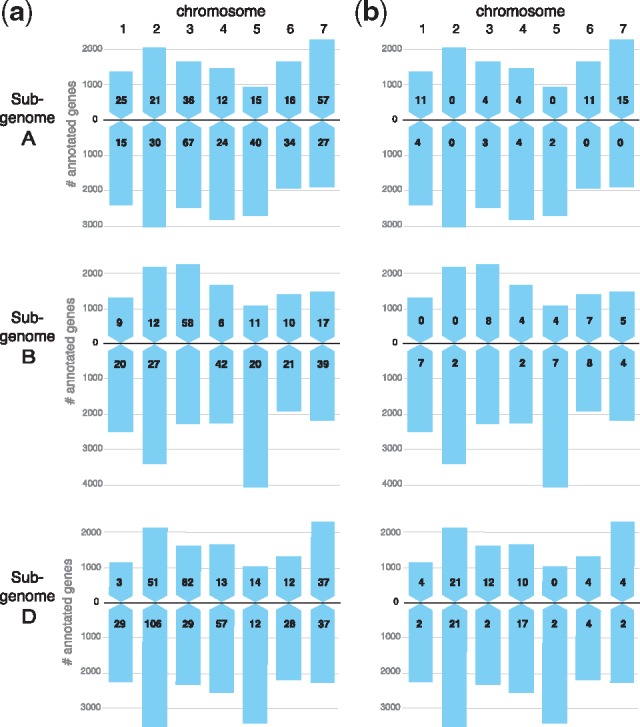
High-confidence inferred gene splits on the wheat genome. A, B and D refer to the three subgenomes of the hexaploid wheat genome. (**a**) Number of unambiguous predictions for each chromosome arm. (**b**) Number of ambiguous predictions (i.e. for which there are more than two candidate fragments for a single juncture). Pairs of fragments are inferred separately for each chromosome arm of flow-sorted *Triticum aestivum* cv. Chinese Spring, except chromosome 3B, for which the analysis was performed on the entire chromosome

Finally, we applied our tests to infer split genes on the rest of the bread wheat genome, i.e. all chromosomes other than 3B. Based on the analyses on simulated fragmentation and between two assemblies (see above), we determined parameters for the tests. For each chromosome arm, we obtained gene families by running OMA GETHOGs with default parameters. In the collapsing approach, we collapsed all branches with bootstrap support less than 0.95, and we performed the likelihood ratio test with the significance level of 0.01. The intersection of predictions identified 1442 pairs in total: 1221 unambiguous and 221 ambiguous cases. The distribution of the number of predictions per chromosome is shown in Figure 3 (see also Supplementary File S3) while fragment IDs are provided in Supplementary file esprit2_predictions_wheat.tar.gz.

## 5 Discussion and outlook

Despite technological and algorithmic advances, genome assembly and annotation remain a challenge, especially for large polyploid genomes with complex evolutionary histories. Genes often remain fragmented and fragments get annotated as separate genes. Our work demonstrates that using available assemblies of related species can provide enough information to recognize some of those cases and obtain full-length genes.

We developed two approaches and showcase their good performance on a challenging proteome of hexaploid bread wheat (*T. aestivum* cv. Chinese Spring). In simulations and validation, both of which were performed on the real data taking into account all its complexities, the approach relying on collapsing gene tree branches showed lower recall and higher precision than a likelihood ratio test (Fig. 2). As a trade-off between precision and recall, we propose taking an intersection of their predictions, as we did in the quest for fragmented genes in the wheat survey sequence dataset. As our stringent simulation and real data assessment shows, the inferred split genes are highly specific. The performance is even better when we combine the new phylogeny-based tests to our earlier pairwise approach ‘ESPRIT’.

The two main inherent challenges of *in silico* split gene inference are the confounding effect of close paralogs and the variation in the rate of evolution along the sequences. Indeed, sometimes fragments come from identical or nearly identical paralogs, and there is not enough information to distinguish fragments belonging to one gene from another. Hence, we are more likely to make a false positive prediction (Supplementary Fig. S6). This was particularly salient in the artificial fragmentation analyses on the cassava genome. Indeed, cassava is known to have undergone a whole-genome duplication around 35–47 MYA ago (Bredeson *et al.*, 2016), which is not shared by another reference species in our analysis. As a result, many pairs of paralogs are specific to this species, and thus virtually indistinguishable from fragments of the same gene by our method.

As for the second main challenge, evolutionary rate heterogeneity across the protein length, this can pose problem because fragments of the same genes can wrongly appear to be coming from distinct sequences. Consider for instance a protein composed of two domains—one slowly evolving and one fast evolving. If we consider each domain as a distinct sequence and look at their position in a gene tree including full-length homologous counterparts, the branch lengths connecting these fragments to the rest of the tree may have markedly different lengths. As a consequence, the increase in likelihood obtained by having distinct branches for each fragment may be sufficiently large for our test to erroneously infer that the fragments come from distinct sequences (see Supplementary Table S5 for an actual example). It may be possible to address this problem by more explicitly modelling variation of rate among sites.

At a practical level, predictions heavily depend on the choice of two parameters: a threshold for collapsing branches and a significance level for the likelihood ratio test. Lower, more stringent thresholds for collapsing yield more confident predictions, while higher, less conserved thresholds will produce more predictions but less confident. Similarly, a higher significance of the likelihood ratio test will result with less but more confident predictions. Obtaining more predictions can be achieved by lowering the significance of the test at the cost of their lower confidence. Overall, it is important to choose thresholds depending on the application. For example, a higher number of predictions can be favourable for comparison with other data.

Predictions also depend on the input families. Bigger gene families facilitate more predictions (Fig. 2, Supplementary Fig. S3) but also result in more ambiguous calls, i.e. cases where a fragment is involved in multiple predictions (Supplementary File S2). We observed fewer false positive predictions when we simulated fragmentation on bigger gene families where we were more likely to randomly split a pair of more distant paralogs in comparison to small gene families which are more likely to contain only very close paralogs (Fig. 2a, Supplementary Fig. S3a). However, the results of validation indicate that the methods are still able to identify a reasonable number of split genes with high precision even when small gene families are used.

Throughout this project, we fixed some of the parameters. First, we considered only genes at least 50 amino-acids long. Shorter sequences contain less information thus make phylogeny reconstruction more challenging; at the same time, the benefit of putting together short fragments is also more limited. Second, we required candidate fragments to overlap less than 10%. Increasing the overlap increases the number of candidate pairs and, consequently, the number of predictions including false positive and ambiguous predictions. Finally, we used Mafft v7.164b to align gene families and FastTree v2.1.8 to reconstruct gene trees, both with their default parameters due to their convenience and speed. Exploring their parameter space or using more suitable tools for the dataset of interest could contribute to higher precision and recall.

As often with new approaches, the likelihood ratio test still has room for improvement. Currently, we compute the distribution of the test statistic empirically, via resampling. We computed up to 500 samples per test which, given the simulations and validation, seems to be enough here; yet the convergence of the distribution could be explored. Increasing the number of samples might lead to significantly better approximation of the distribution and more accurate results. In addition, parameterizing the distribution of the test statistic would reduce computational time and memory usage.

Since both tests rely on evolutionary relationships, some of the mistakes could be avoided by implementing a more realistic evolutionary model. This is of particular importance for cases which are missed due to differences in evolutionary rates across the length of the gene.

To further improve the performance, one could try to find optimal parameters for the dataset of interest and application in question. Different strategies could be used to obtain input families as well as alternative tools for alignments and methods with more exhaustive optimal tree search. For datasets with relatively close levels of divergence, tree inference based on nucleotide instead of amino-acid sequences might confer more statistical power to our tests. It may also be possible to exploit transcriptome data as additional source of information (Zhang *et al.*, 2016).

But already in its present form, as the large number of detected split genes in the wheat genome illustrates, our approach is already proving highly useful. All computer code is available for reuse as a user-friendly package named ‘ESPRIT 2.0’ (https://github.com/DessimozLab/esprit2) that we hope will help make phylogeny-based detection of split genes a routine step in genome assembly and annotation.

## Supplementary Material

Supplementary Data
