# Supplementary material for: Phylogenetic approaches to identifying fragments of the same gene, with application to the wheat genome
Source: Bioinformatics. 2018 Sep 1;35(7):1159–66. doi: 10.1093/bioinformatics/bty772 (PMC6449756; doi:10.1093/bioinformatics/bty772)
Supplement: Supplementary Data [file bty772_supp.zip › bty772-Suppl_data/bty772_Supplementary.pdf]

Supplementary materials

Phylogenetic approaches to identifying fragments of the same gene, with application to the wheat genome

Ivana Piližota et al.

Implementation of the tests

Input data

Concatenating non-overlapping fragments into a single sequence

Concatenation was done at the level of multiple sequence alignment, i.e., we aligned a gene family and then replaced the two fragments with a newly created sequence containing residues from both fragments and gaps at the remaining positions.

Working with overlapping fragments

Two candidate fragments were allowed to overlap for less than *10%* of the region length each of them spanned in the multiple sequence alignment. Looking at the alignment from left to right, this means that the end of one fragment and the beginning of the other overlap. We do not attempt to resolve any potential mismatch in the overlapping region. Instead, we first determine the *middle* of the overlap and edit the sequences as follows: in the sequence on the left, we kept all positions the same up to the *middle* and replaced the remaining residues with X's. Similarly, in the sequence on the right, we edited the beginning of the sequence by replacing all residues up to the *middle* with X's and then kept all remaining residues as they were. This way we got two fragments with non-overlapping known residues in the alignment. They were used as such in both tests. For trees under the H_s model in the likelihood ratio test, these modified sequences were concatenated as described in the section on concatenating non-overlapping fragments (see above).

Starting topology for H_p model

As a starting topology in the search for the maximum likelihood tree under the H_p model, we used a modified tree from the H_s model. We bifurcate the leaf with the candidate split gene and set the new branches' lengths to zero (Suppl. fig. 1). Since heuristics for the tree search can get stuck in a local optimum, this is to provide a starting point with a likelihood at least equal to that of H_s. Indeed, since gaps are treated as missing data, the likelihood is computed by integrating over every possible state at each gap position (this is referred to as the "marginal likelihood").

Since the number of observed characters is the same under H_p *and* H_s, aligned in the same way, the marginal likelihoods are identical.

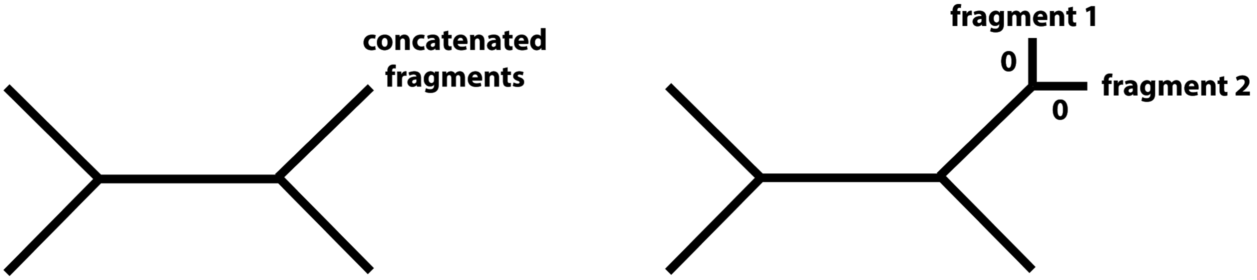

Supplementary figure 1: Input topology for the likelihood ratio test. Left: Maximum likelihood tree under the H_s. Right: Modified tree to be used as a starting point in the optimisation of the tree in the H_p model.

Datasets and evaluation methodology

Random fragmentation of the wheat 3B reference assembly (recall)

Fragmentation

First, we aligned corresponding gene families using Mafft v7.164b with default settings. Then we chose a random position *n* in the alignment such that the target gene, i.e., the one we want to fragment, contains at least 50 amino-acids in the first *n* positions of the alignment and at least 50 amino-acids right of the chosen position. The first *n* positions of the aligned target gene extended by *alignment_length-n* gaps form one fragment, while the second fragment is formed from *n* gaps extended by the rest of the aligned target sequence. The original target sequence is then replaced with the newly formed fragments while the rest of the gene family is kept the same (Suppl. fig. 2a).

Simulations on HOGs

We performed the experiment as follows:

1) We computed HOGs (input data in Suppl. table 1) using the GETHOGs algorithm implemented in OMA standalone, keeping default settings.
2) A random 3B gene was selected, and a HOG at the deepest taxonomic level (“root-level HOG”) containing the gene was aligned using Mafft v7.164b (default settings). The gene was split at a random position (see above).
3) All necessary trees were computed using default settings in FastTree v2.1.8.
4) Both methods were applied to all pairs of candidates.

Introducing non-overlapping paralogs in wheat 3B reference assembly (precision)

Fragmentation

Again, we aligned gene families using Mafft v7.164b with default settings. Genes from a randomly chosen pair of paralogs were assigned to *sequence 1* and *sequence 2* at random. Then we chose a random *n* such that the first *n* positions of *sequence 1* and last *alignment_length-n* positions of *sequence 2* each contain at least 50 amino acids. These two subsequences form the basis of simulated fragments, one extended by gaps on its right end and the other extended by gaps at its left end (Suppl. fig. 2b). If there was no such *n*, the pair was discarded.

Simulations on HOGs

Analogous to the *Simulations on HOGs* in *Random fragmentation of the wheat 3B reference assembly (recall)*.

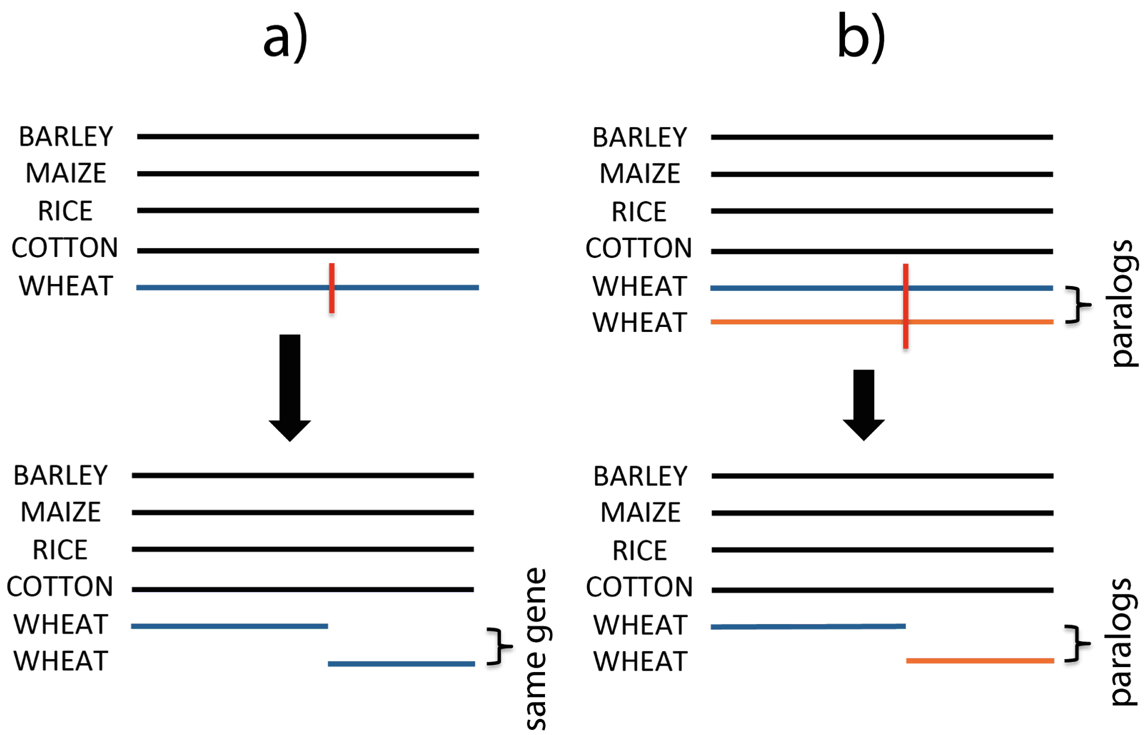

Supplementary figure 2: Simulating fragmentation. a) Simulating fragments coming from the same gene, b) Simulating fragments coming from paralogs.

Species	Database
Aegilops tauschii	Ensembl Plants 21
Arabidopsis thaliana	Ensembl Plants 20
Brachypodium distachyon	Ensembl Plants 21
Hordeum vulgare var. distichum	Ensembl Plants 16
Oryza brachyantha	Ensembl Plants 21
Oryza glaberrima	Ensembl Plants 21
Oryza sativa subsp. indica	Ensembl Plants 21
Oryza sativa subsp. japonica	Ensembl Plants v7
Setaria italica	Ensembl Plants 21
Sorghum bicolor	Sbi1_4
Triticum aestivum cv. Chinese Spring	Ensembl Plants 26
Triticum urartu	Ensembl Plants 19
Zea mays	Ensembl Plants v8

Supplementary table 1: Proteomes exported from the OMA Browser and used as input data for the GETHOGs algorithm in simulations on wheat. The second column contains information on the database release from which the assembly and annotation is from.

Introducing fragmentation in cassava assembly (recall, precision)

Fragmentation

Analogous to Fragmentation in *Random fragmentation of the wheat 3B reference assembly (recall)* and *Introducing non-overlapping paralogs in wheat 3B reference assembly (precision).*

Simulations on HOGs

We performed the experiment as follows:

1) We computed HOGs (input data in Suppl. table 2) using the GETHOGs algorithm implemented in OMA standalone, keeping default settings.
2) We filtered out “root-level” HOGs containing no cassava genes, more than 100 genes (in total, from any species) or less than 9 reference species.

3) [Simulating fragments coming from the same gene] A random cassava gene (at least 200 amino-acids long) was selected, and a “root-level” HOG containing the gene was aligned using Mafft v7.164b (default settings). The gene was split at a random position (see above).
   [Simulating fragments coming from paralogs] A random pair of cassava paralogs (each at least 200 amino-acids long) was selected, and a “root-level” HOG containing the pair was aligned using Mafft v7.164b (default settings). The genes were split at a random position (see above).
4) All necessary trees were computed using default settings in FastTree v2.1.8.
5) Both methods were applied to all pairs of candidates; collapsing with threshold 0.95 and LRT with significance 0.01. We took the intersection of their predictions.

Species	Database
Arabis alpina	ENA
Arabidopsis lyrata	Ensembl 37
Arabidopsis thaliana	Ensembl Plants 20
Brassica napus	Genoscope
Brassica oleracea	Ensembl Plants 23
Brassica rapa subsp. pekinensis	Ensembl Plants 36
Gossypium hirsutum	OMA Browser, Dec 2017
Lotus japonicus	modENCODE
Manihot esculenta (v4.1)	Phytozome 7
Medicago truncatula	Ensembl Plants 18
Populus trichocarpa	Ensembl Plants 15
Prunus persica	Ensembl Plants 23
Solanum lycopersicum	Ensembl Plants 27
Solanum tuberosum	Ensembl Plants 21
Glycine max	Ensembl Plants 19
Theobroma cacao	Ensembl Plants 23
Vitis vinifera	Ensembl Plants v9

Supplementary table 2: Proteomes exported from the OMA Browser and used as input data for the GETHOGs algorithm in cassava simulations. The second column contains information on the database release from which the assembly and annotation is from.

Validation on 3B survey assembly

**Species**	**Database**
Aegilops tauschii	Ensembl Plants 21
Arabidopsis thaliana	Ensembl Plants 20
Brachypodium distachyon	Ensembl Plants 21
Hordeum vulgare var. distichum	Ensembl Plants 16
Oryza brachyantha	Ensembl Plants 21
Oryza glaberrima	Ensembl Plants 21
Oryza sativa subsp. indica	Ensembl Plants 21
Oryza sativa subsp. japonica	Ensembl Plants 27
Setaria italica	Ensembl Plants 21
Sorghum bicolor	Sbi1_4
Triticum aestivum cv. Chinese Spring	Ensembl Plants 21
Triticum urartu	Ensembl Plants 19
Zea mays	Ensembl Plants 27

Supplementary table 3: Proteomes exported from the OMA Browser and used as input data for the GETHOGs algorithm in validation on *Triticum aestivum* cv. Chinese Spring chromosome 3B. The second column contains information on the database release from which the assembly and annotation is from.

Procedure

We performed the experiment as follows:

1) We computed HOGs using the GETHOGs algorithm implemented in OMA standalone, keeping default settings. Since OMA requires sequences to be longer than a certain threshold, in order to consider shorter sequences in the target wheat genome, we renamed WHEAT.fa file in OMA/Cache to WHEAT.contig.fa. Indeed, OMA usually requires alignments between homologs to cover a certain proportion of the sequence lengths, to avoid partial matches. For protein sequences defined in genomes with the special “contig.fa” extension, OMA does not apply the alignment coverage criterion.

2) Each top-level HOG was aligned using Mafft v7.164b (default settings) and searched for candidate fragments. Two fragments were called a pair of candidates if their overlaps were below 0.1, i.e., 10%.
3) All necessary trees were computed using default settings in FastTree v2.1.8.
4) Both tests were done on all pairs of candidates.

Control

Since the GETHOGs algorithm was not developed for the purpose of surveying genome assemblies, its default parameters might not be optimal for this goal. In particular, a set of default parameters might be too conservative so we repeated the experiment described above but with some parameters lower than the default (`MinScore := 150, LengthTol := 0.4, ReachabilityCutoff := 0.3`). This yielded bigger HOGs and hence more candidates to test.

BLAST+ validation

We developed two modes of BLAST+ validation. A less stringent one conditions only on query coverage per hit (`qcovs`) and %identical matches (`pident`). A more stringent one allows mismatches to be only at the ends of a query sequence.

Less stringent validation

For each unambiguous or ambiguous prediction we:

1) Take initial non-modified sequences of both fragments and BLAST+ them against the high-quality assembly of chromosome 3B (`-evalue 0.001`)
2) For each query sequence, identify BLAST+ hit(s) with the highest bitscore (`bitscore`). Keep only hit(s) with `qcovs` >= 95 and `pident` >= 95, if any. If there are no such hits for any of the queries, the pair cannot be validated.
3) For each query and its hits from 2., keep only hits with the highest `qcovs`. If there are multiple hits per query that satisfy the criteria, then filter out all hits with `pident` lower than the highest present. If there are still multiple hits for any of the queries, we consider it an ambiguous mapping and do not validate the pair.
4) If both queries have the same best hit, the prediction is considered to be correct. Otherwise, we consider it wrong.

More stringent validation

All steps are the same as in *Less stringent validation* except the step 2. Here, in addition to `qcovs` >= 95 and `pident` >= 95, we require all mismatches between a query and a hit to be at the ends of a query sequence.

Let's say that our tolerance length is M. Suppose that first N_1 and last N_2 positions of a query are not covered by a hit. If $N_1 > M$ or $N_2 > M$, then the hit does not pass the criteria. For a given

query and a hit such that $0 <= N_1, N_2 <= M$, consider their BLAST+ alignment. We allow mismatches to be only in the query's first $M-N_1$ or last $M-N_2$ aligned positions, and we set M=5.

Results

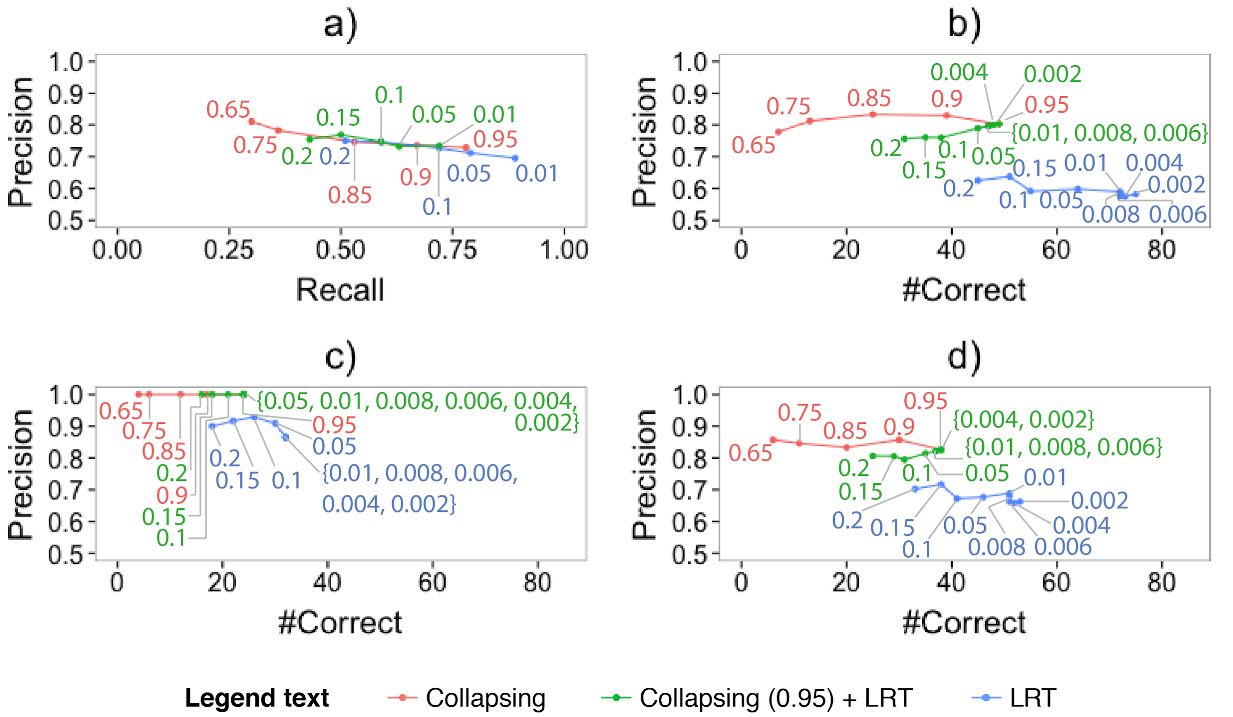

Supplementary figure 3: Results of control experiments: simulations and validations. a) Simulated fragmentation on HOGs with default settings in the GETHOGs algorithm, b) Validation on the 3B survey sequence using HOGs with relaxed parameters in the GETHOGs algorithm, less stringent BLAST+ validation, c) Validation on the 3B survey sequence using HOGs with default settings in the GETHOGs algorithm, more stringent BLAST+ validation, d) Validation on the 3B survey sequence using HOGs with relaxed parameters in the GETHOGs algorithm, more stringent BLAST+ validation.

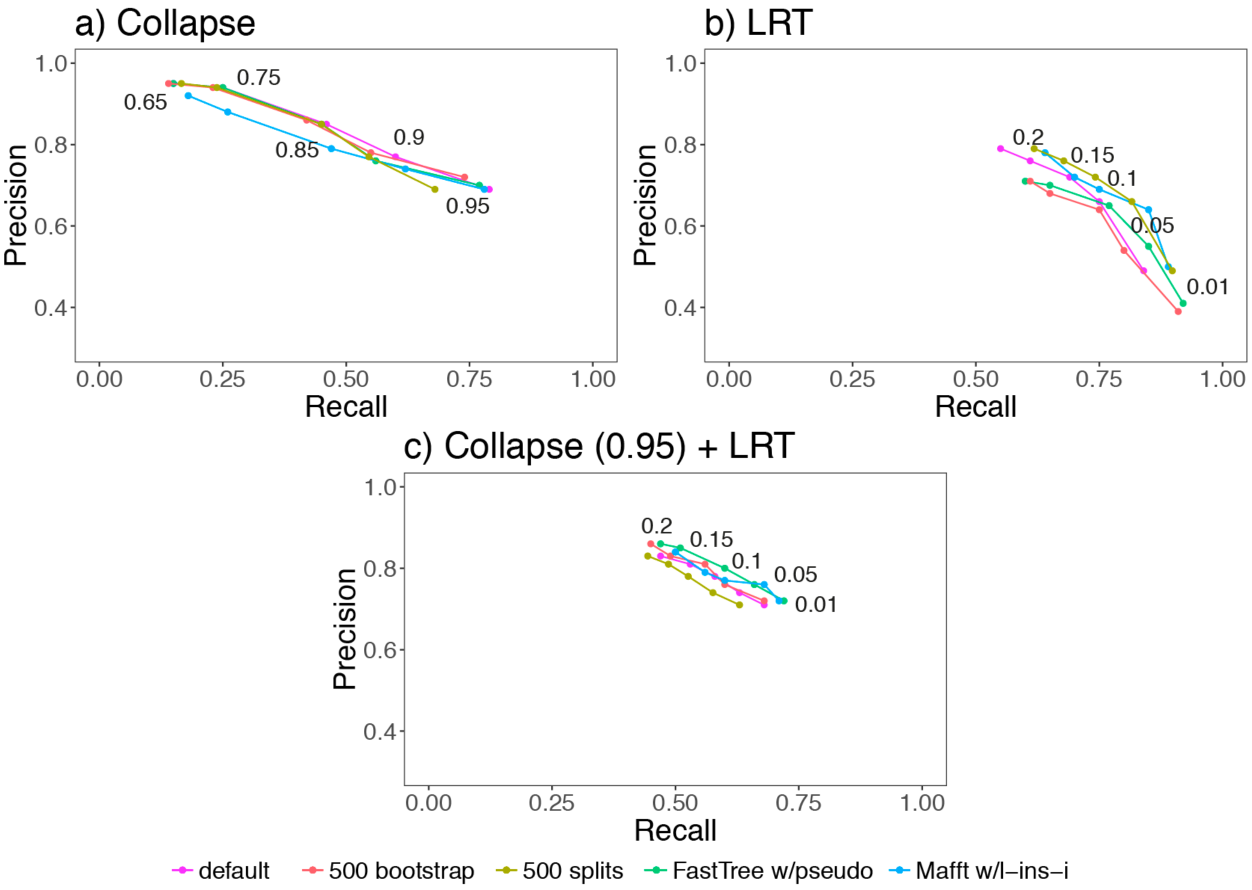

Supplementary figure 4: Various parameter variations have little impact on our phylogenetic tests. We compared the default pipeline (pink) with using 500 bootstrap replicates instead of 100 in the LRT test (red), introducing 500 artificial splits instead of 100 (beige), using the FastTree option "-pseudo" for fragmentary genes (green) and using Mafft L-INS-I instead of FFT-NS-2 (blue).

Comparison to other methods

Summary of predictions on the data that can be found in OMA				
	#correct	#wrong	#could not validate	#total
Ensembl Compara	33	6	47	86
ESPRIT	55	3	146	204
Meta-method based on ESPRIT and the two new tests	73	3	166	242
Summary of all Ensembl's unambiguous predictions				
	#correct	#wrong	#could not validate	#total
	43	6	57	106

Supplementary table 4: Comparison to Ensembl Compara, ESPRIT, and meta-method.

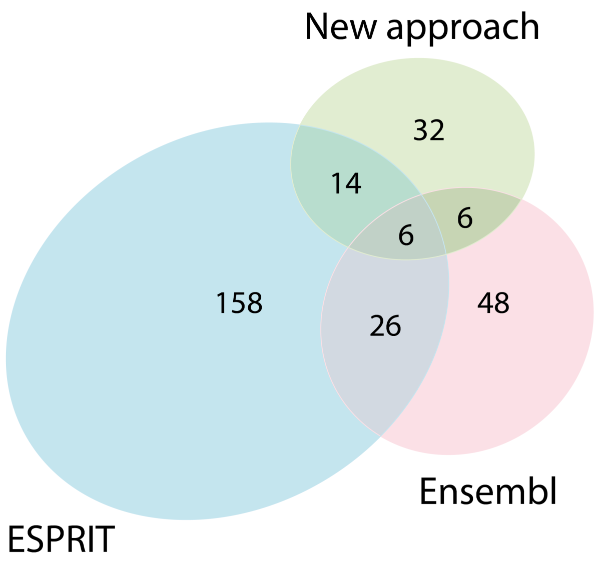

Supplementary figure 5: Comparison to Ensembl Compara and ESPRIT. The number of predictions inferred by each method on the 3B survey sequence—also including predictions that could not be validated due to ambiguous mapping between the survey and reference wheat 3B assemblies. The “new approach” combines Collapsing (threshold 0.95) and LRT (significance 0.01).

Discussion

Mistakes in the likelihood ratio test

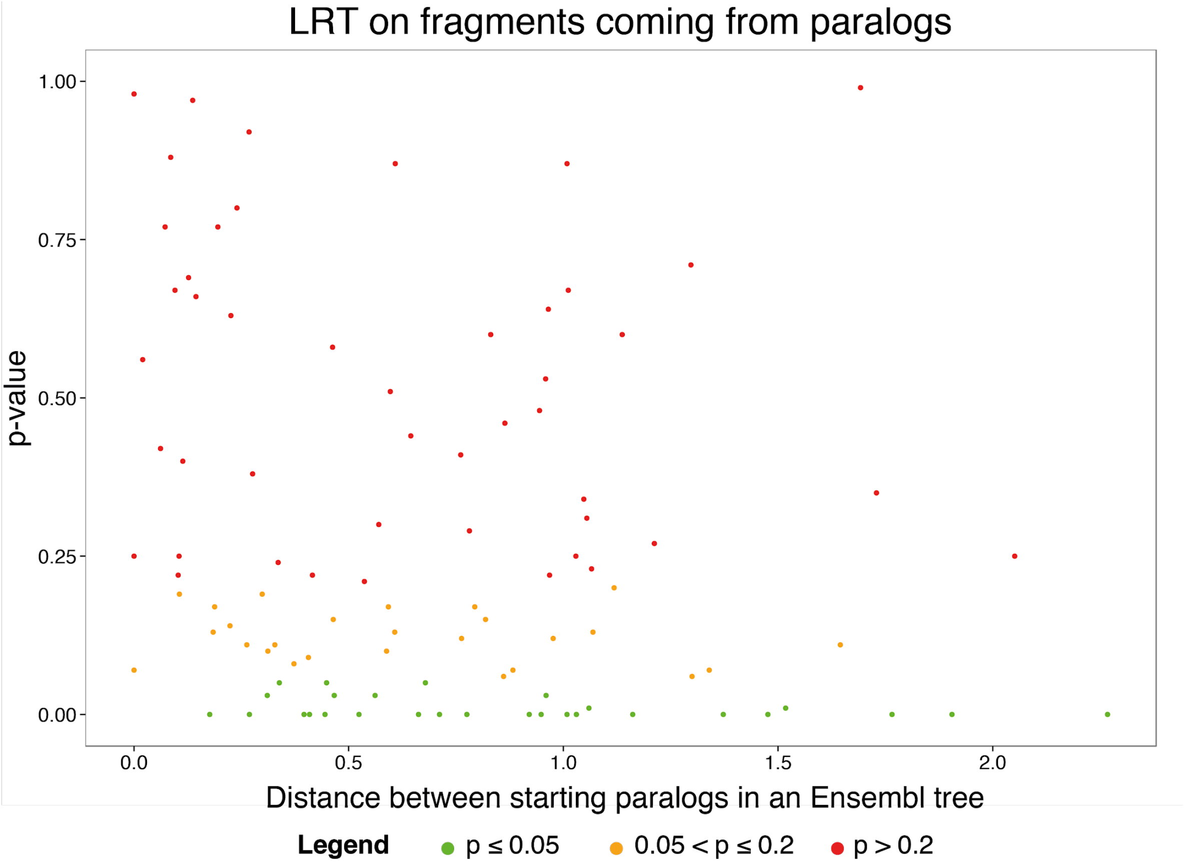

Supplementary figure 6: The relationship between the paralog distance (expected number of changes per site) and the p-value for the likelihood ratio test when applied to random fragments coming from the paralogs.

Fragments coming from regions that have evolved at different rates

Gene TRAES3BF091400260CFD_t1 was split at a random position. The p-value of the likelihood ratio test is probably low, i.e. in favour of the hypothesis that fragments come from paralogous sequences, due to dissimilar evolutionary rates. The collapsing approach correctly infers them as fragments of the same gene when used with any of the thresholds {0.65, 0.75, 0.85, 0.9, 0.95} (Suppl. table 5).

The gene family was obtained from Ensembl Plants, release 31, alignments performed by Mafft v7.164b (Suppl. File alignments.tar.gz) and trees built with FastTree v2.1.8 (Suppl. fig. 7).

Gene	P-value	#references in the MSA	Length of the MSA	Length (gene)<br><br>Length (fragment1)<br><br>Length (fragment2)	Results from collapsing approach
TRAES3BF091400260CFD_t1	0.02	3	355	244<br><br>92<br><br>152	Split gene

Reference sequence	PAM distance (reference, fragment1)	PAM distance (reference, fragment2)
TRIUR3_21111-P1	43.06	734

Supplementary table 5: Information on a case where the likelihood ratio test distinguishes fragments coming from the same gene.

a) b)

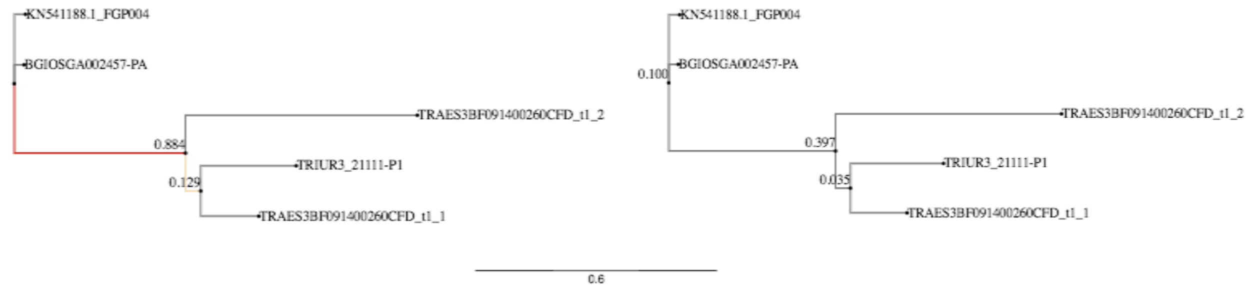

Supplementary figure 7: Gene tree containing fragments of the TRAES3BF091400260CFD_t1 gene. a) With branch bootstrap supports, b) With branch lengths.

Mistakes on distant sequences

To illustrate more cases where the likelihood ratio test makes incorrect inference, we also provide information on three cases (Suppl. table 6, alignments in Suppl. File esprit2_alignments.tar.gz). In all of them, we introduced fragmentation on distant paralogous sequences which should be sufficient to distinguish fragments as paralogous. Yet, based on the p-values and levels of significance of the test, we could not reject the hypothesis that fragments come from the same gene.

Given the size of the gene families, non-conserved long alignments and rather short candidate fragments, there is likely the lack of information contained in the alignments and fragments to

make a correct inference. However, there is enough information for the collapsing approach to infer that pairs in question are paralogous.

Gene1	Gene2	Distance_in_Ensembl_tree (gene1, gene2)	P-value	#genes in the MSA	Length of the MSA	Length (gene1)<br><br>Length (fragment 1)	Length (gene2)<br><br>Length (fragment2)	Results from collapsing approach
TRAES3BF071900030CFD_t1	TRAES3BF182800010CFD_t1	2.05	0.25	1040	11165	464<br><br>366	1160<br><br>83	paralogs
TRAES3BF117700060CFD_t1	TRAES3BF136700020CFD_t1	1.73	0.35	1293	16234	554<br><br>140	368<br><br>181	paralogs
TRAES3BF075600050CFD_t1	TRAES3BF171600010CFD_t1	1.69	0.99	1258	4924	243<br><br>81	207<br><br>109	paralogs

Supplementary table 6: Information on three cases where the likelihood ratio test fails to distinguish paralogous sequences.

Datasets

The datasets referenced in this document and the main body (simulated splits, comparison, predictions, case studies, etc.) can be downloaded from http://lab.dessimoz.org/18_esprit2.
